# Cytokine Indicators Associated with Disease Severity in Severe Fever with Thrombocytopenia Syndrome: A Systematic Review and Meta-Analysis

**DOI:** 10.3390/pathogens15070755

**Published:** 2026-07-17

**Authors:** Yaqi Xie, Quanman Hu, Shuaiyin Chen, Baoqin Zhang

**Affiliations:** 1 Zhengzhou Center for Disease Control and Prevention, Zhengzhou 450001, China; yaqixie2022@163.com; 2 Department of Epidemiology and Health Statistics, College of Public Health, Zhengzhou University, No. 100-Kexue Avenue, Zhengzhou 450001, China; quanmanhu@163.com

**Keywords:** severe fever with thrombocytopenia syndrome, cytokine indicators, severe cases, mild cases, meta-analysis

## Abstract

Objective: The purpose of this study is to study cytokine indicators for the identification of severe fever with thrombocytopenia syndrome (SFTS) severity. Methods: We searched the literature in PubMed, Embase, and Web of Science published before 7 April 2026. The main results are presented as forest plots. Subgroup analyses, sensitivity analyses, and publication bias were also performed. Results: A total of 22 articles were eventually included in our study. Our findings demonstrate that circulating concentrations of Interleukin-6 (IL-6) (SMD = 2.02, 95% CI: 1.53–2.51, I^2^ = 95.2%), Interleukin-10 (IL-10) (SMD = 1.18, 95% CI: 0.92–1.44, I^2^ = 71.3%), Interleukin-8 (IL-8) (SMD = 0.91, 95% CI: 0.61–1.20, I^2^ = 69%), Tumor necrosis factor-alpha (TNF-α) (SMD = 0.70, 95% CI: 0.43–0.96, I^2^ = 64.6%), Interferon-gamma (IFN-γ) (SMD = 1.32, 95% CI: 0.69–1.95, I^2^ = 89.1%), Interleukin-1 beta (IL-1β) (SMD = 1.78, 95% CI: 0.85–2.71, I^2^ = 94.8%), Monocyte chemoattractant protein-1 (MCP-1) (SMD = 1.15, 95% CI: 0.80–1.50, I^2^ = 46.1%), Interferon-alpha (IFN-α) (SMD = 1.53, 95% CI: 0.38–2.68, I^2^ = 89.6%), Granulocyte Colony-Stimulating Factor (G-CSF) (SMD = 1.79, 95% CI: 0.99–2.59, I^2^ = 68.1%) and Inducible protein 10 (IP-10) (SMD = 1.11, 95% CI: 0.60–1.62, I^2^ = 58.3%) are significantly elevated in patients with severe SFTS compared with those with mild disease, whereas Transforming Growth Factor-beta (TGF-β) (SMD = −0.51, 95% CI: −0.78–−0.24, I^2^ = 6.0%) and RANTES (SMD = −0.10, 95% CI: −0.40–0.20, I^2^ = 0.0%) levels are reduced in the severe group. Conclusions: By analyzing the cytokine indicators of SFTS patients, we have found some indicators that are representative of SFTS severity. Our findings provide a clinically actionable basis for early severity prediction and further useful evidence for clinicians to manage severe patients efficiently.

## 1. Introduction

Severe fever with thrombocytopenia syndrome (SFTS) is an acute infectious illness transmitted by ticks and triggered by the SFTS virus (SFTSV) [1]. The disease was first identified in 2009 in rural central China, specifically in the Dabie Mountains region [2]. At that time, a novel sandfly virus was isolated from the blood of a patient initially suspected of being infected with human granulocytic anaplasmosis, later named SFTSV [3]. SFTSV is a single-stranded RNA virus, and it exhibits broad host tropism, infecting humans, domestic livestock, and wild reservoir hosts, thereby sustaining enzootic cycles and enabling spillover into human populations [4,5]. SFTS cases are predominantly reported from rural mountainous and hilly regions of East and Central Asia, with epidemiological patterns characterized by sporadic occurrence and seasonal peaks during late spring through early autumn [6,7,8]. All age groups are susceptible, though incidence is significantly higher among adults aged ≥50 years engaged in outdoor agricultural or forestry activities [9,10]. Clinical presentation typically includes abrupt-onset fever, marked thrombocytopenia, leukopenia, gastrointestinal symptoms, neurological manifestations, and progressive multi-organ dysfunction. In severe cases, liver damage, renal impairment, coagulation disorders, and respiratory distress may occur [11,12]. In addition to typical symptoms, complications such as myocardial dysfunction and neurological disorders may also occur [13,14]. Approximately 37.5% of confirmed cases of SFTS progress to severe illness, with a case fatality rate as high as 30% among severe cases [15].

Given the absence of approved antiviral agents and the high morbidity and mortality associated with SFTS, a range of therapeutic strategies have been empirically investigated in clinical practice and research settings. Although Lee et al.’s study demonstrated that ribavirin could reduce SFTSV replication and cytopathic effects [16], several other investigations have reported no significant difference in mortality rates between patients receiving ribavirin and those in the control group, and ribavirin did not significantly lower viral load or improve thrombocytopenia regardless of disease severity [17,18,19]. Favipiravir (T-705) has demonstrated promising antiviral activity in preclinical investigations, but its efficacy in severe patients still requires further evidence [20]. Although both glucocorticoids and intravenous immunoglobulin (IVIG) can suppress systemic inflammation and alleviate the resulting cytokine storm, their use has remained controversial [21,22,23]. In a case report study, therapeutic plasma exchange (TPE) alleviated the cytokine storm and reduced viral load in the early stages of SFTS [24], indicating that TPE is a promising treatment option. Notably, tocilizumab is a humanized monoclonal antibody designed to bind the interleukin-6 (IL-6) receptor, thereby selectively inhibiting downstream IL-6 signal transduction [25]. IL-6 functions as a central pro-inflammatory cytokine implicated in the cytokine storm observed in SFTS [26]. Clinical evidence indicates that tocilizumab administration is associated with a marked decrease in patient fatality [25,26,27].

This prompts us to consider that current treatments for SFTS patients remain highly controversial, and cytokine storm appears to be a central issue faced by severe SFTS cases. Some studies have focused on anti-cytokine therapies, such as tocilizumab, an agent targeting the IL-6 pathway [26,27]. Therefore, we focused on the levels of cytokines in SFTS patients at different disease stages, aiming to clarify the impact of cytokine levels on the progression of SFTS, and to provide scientific evidence and insights for identifying high-risk factors and developing therapeutic strategies.

## 2. Materials and Methods

### 2.1. Literature Search Strategy

We conducted a systematic search across PubMed, Embase, and Web of Science covering all published literature as of 7 April 2026. The search terms used were: “Severe Fever with Thrombocytopenia Syndrome” OR “SFTSV” OR “SFTS” and “Cytokines” OR “Chemokine” OR “Interleukin”. Full search strings for each database are detailed in Table S1. The articles selected for this study were identified by conducting an extensive review of existing systematic reviews and meta-analyses, thereby maximizing coverage of pertinent scholarly work. However, non-peer-reviewed sources were deliberately excluded. Thereafter, titles and abstracts of retrieved records were independently screened by two reviewers for relevance, and full-text articles meeting inclusion criteria were obtained for standardized data extraction.

### 2.2. The Criteria for Inclusion and Exclusion

The selected studies had to meet the following eligibility requirements: (1) study participants were laboratory-confirmed SFTS patients. (2) The study reported the levels of cytokine markers in patients with mild and severe SFTS. (3) The study included clinical symptoms of SFTS and cytokine levels. (4) Patients in the study were divided into mild versus severe groups, or survival versus death groups.

The exclusion criteria for articles are as follows: (1) systematic reviews and meta-analyses, letters, and commentaries; (2) incomplete cytokine data or lack of precise numerical values in the article; and (3) cytokine outcome results presented in non-numeric formats.

### 2.3. Definition of Mild and Severe Cases

Mild cases of SFTS may present symptoms such as fever, thrombocytopenia, loss of appetite, dizziness, headache, nausea, vomiting, and diarrhea; whereas severe SFTS patients may develop multiple organ dysfunction syndrome, including encephalopathy, heart failure, acute respiratory distress syndrome, severe pancreatitis, cytokine storm, acute kidney injury (AKI), and disseminated intravascular coagulation [14,28,29,30,31,32,33,34,35,36].

### 2.4. Literature Screening and Data Extraction

This study followed the requirements of the PRISMA 2020 statement guidelines (Table S2), with registration number CRD420261413450 (https://www.crd.york.ac.uk/PROSPERO/home, accessed on 3 June 2026). Data screening and extraction were conducted independently by two authors, who applied a pre-designed, standardized extraction template developed specifically for this study. Discrepancies arising during quality evaluation, data extraction, or interpretation were settled via consensus reached through mutual discussion. The methodological quality of all eligible studies was evaluated using the Newcastle-Ottawa Scale (NOS); in cases of disagreement, a third author was consulted to reach a final decision. A score of ≥7 on the NOS was considered high quality, 5–7 as moderate quality, and <5 as low quality. Data retrieved from each included study encompassed the following items: source journal, author(s), study year, study duration, sample size, mean age, gender distribution, case numbers (mild vs. severe group or survivors vs. non-survivors), cytokine indicators, and NOS scores.

### 2.5. Statistical Analysis

For studies presenting data as mean ± standard deviation, we used random-effects or fixed-effects models to calculate the standardized mean difference (SMD) estimates and their 95% confidence intervals (CI). If a study did not report data in the mean ± standard deviation format, we converted the provided median (interquartile range) into mean ± standard deviation using the Tong transformation method [37,38] to estimate the SMD and 95% CI, and detailed conversion formulas are available in Table S7. The I^2^ statistic was used to assess heterogeneity among included studies, with significant heterogeneity indicated when I^2^ exceeded 50% or *p* < 0.05. For every outcome assessed, a random-effects model was used if significant heterogeneity was observed; otherwise, a fixed-effects model was applied. Additionally, subgroup analyses were performed to investigate possible contributors to heterogeneity based on patient groups (mild vs. severe cases, survivors vs. non-survivors), mean age, sex ratio, sample size, study period, and study time. Finally, Begg’s test and Egger’s test were employed to evaluate publication bias, and sensitivity analyses were performed to assess the robustness of the results. All statistical analyses were carried out in Stata version 11.0 (Stata Corporation, College Station, TX, USA) with a significance level set at α = 0.05.

## 3. Results

### 3.1. Study Selection and the Characteristics of Included Studies

**Figure 1:**
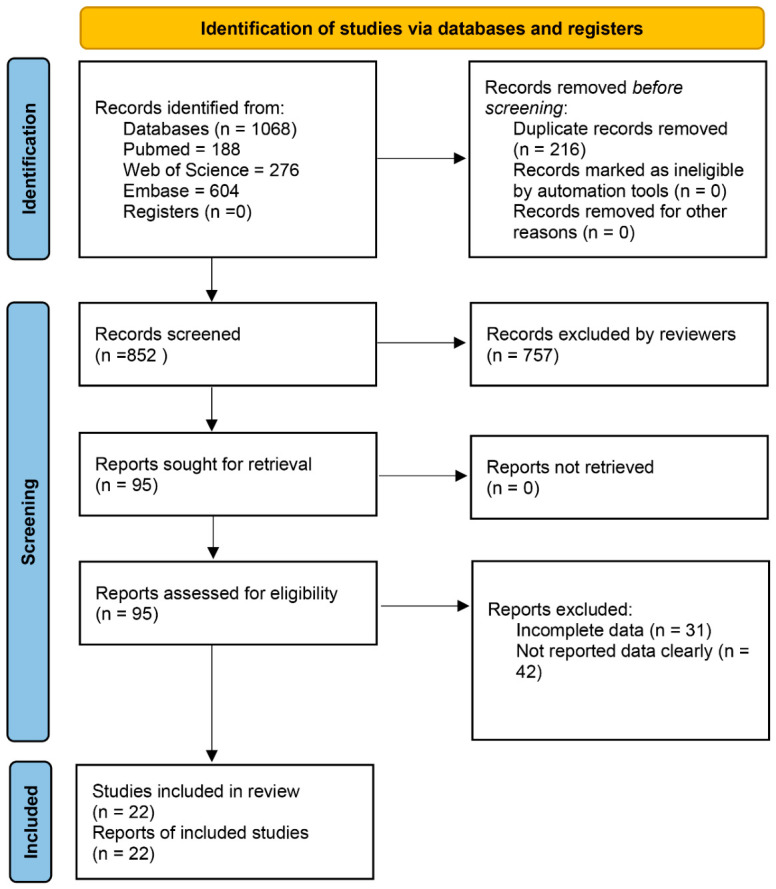
Flowchart showing the screening process for included articles.

We retrieved 1068 records, which were reduced to 852 after removing duplicates. Following a review of titles and abstracts, 757 records were excluded. Finally, after full-text screening of the leftover studies, 22 studies were included in the analysis. The detailed selection process is shown in Figure 1. The sample sizes of the included studies ranged from 10 to 290. The study periods spanned from 2010 to 2024. The proportion of male participants ranged between 30.00% and 72.50%. NOS scores for the included articles ranged from 5 to 9. Key features of the studies included in this analysis are summarized in Table 1.

### 3.2. Effect Estimates

**Figure 2:**
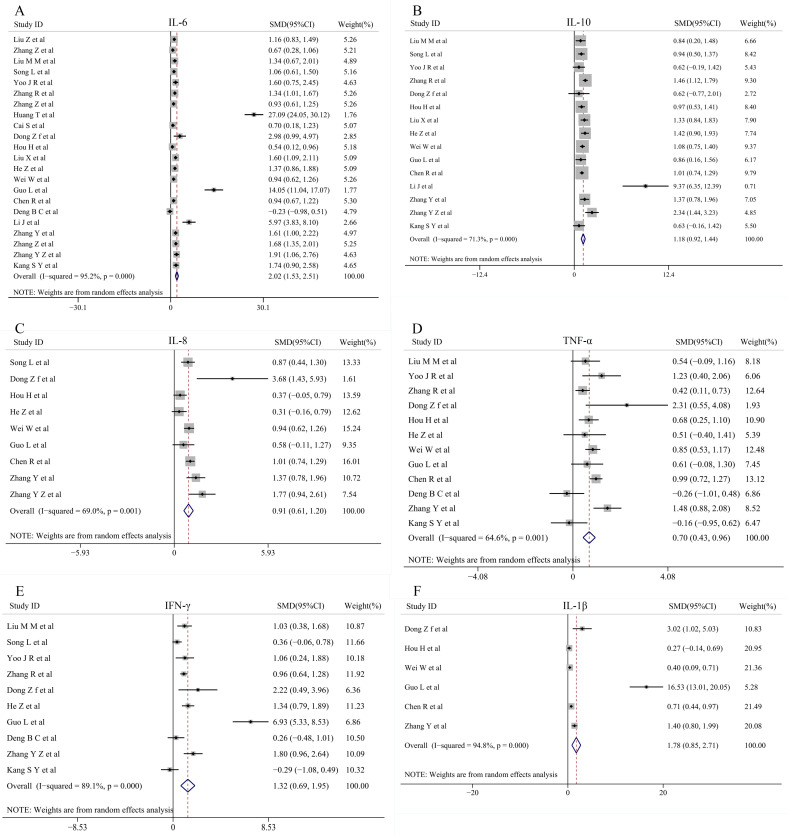
Forest plot for the association of cytokines with SFTS severity. (**A**) (IL-6), (**B**) (IL-10), (**C**) (IL-8), (**D**) (TNF-α), (**E**) (IFN-γ) and (**F**) (IL-1β).

**Figure 3:**
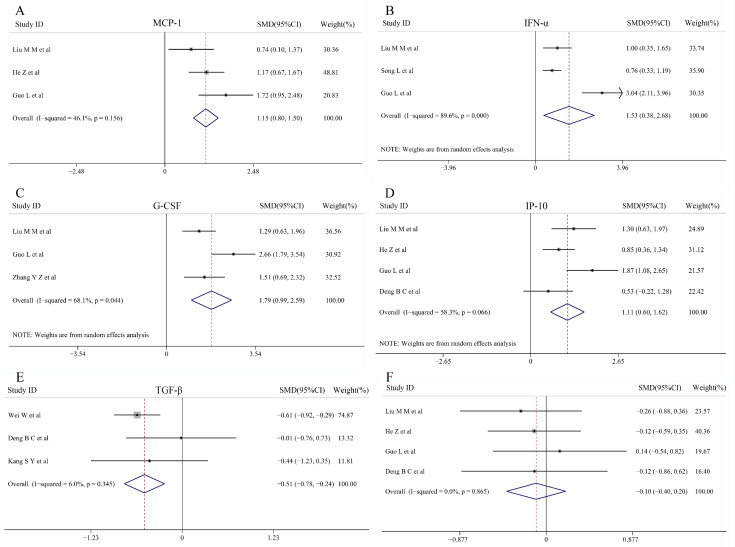
Forest plot for the association of cytokines with SFTS severity. (**A**) (MCP-1), (**B**) (IFN-α), (**C**) (G-CSF), (**D**) (IP-10), (**E**) (TGF-β), and (**F**) (RANTES).

After a comprehensive statistical analysis, we ultimately included the following cytokine indicators: IL-6 (SMD = 2.02, 95% CI: 1.53–2.51, I^2^ = 95.2%, Figure 2A), Interleukin-10 (IL-10) (SMD = 1.18, 95% CI: 0.92–1.44, I^2^ = 71.3%, Figure 2B), Interleukin-8 (IL-8) (SMD = 0.91, 95% CI: 0.61–1.20, I^2^ = 69.0%, Figure 2C), Tumor necrosis factor-alpha (TNF-α) (SMD = 0.70, 95% CI: 0.43–0.96, I^2^ = 64.6%, Figure 2D), Interferon-gamma (IFN-γ) (SMD = 1.32, 95% CI: 0.69–1.95, I^2^ = 89.1%, Figure 2E), Interleukin-1 beta (IL-1β) (SMD = 1.78, 95% CI: 0.85–2.71, I^2^ = 94.8%, Figure 2F), Monocyte chemoattractant protein-1 (MCP-1) (SMD = 1.15, 95% CI: 0.80–1.50, I^2^ = 46.1%, Figure 3A), Interferon-alpha (IFN-α) (SMD = 1.53, 95% CI: 0.38–2.68, I^2^ = 89.6%, Figure 3B), Granulocyte colony-stimulating factor G-CSF (SMD = 1.79, 95% CI: 0.99–2.59, I^2^ = 68.1%, Figure 3C, Inducible protein 10 (IP-10) (SMD = 1.11, 95% CI: 0.60–1.62, I^2^ = 58.3%, Figure 3D), Transforming Growth Factor-beta (TGF-β) (SMD = −0.51, 95% CI: −0.78–−0.24, I^2^ = 6.0%, Figure 3E), RANTES (SMD = −0.10, 95% CI: −0.40–0.20, I^2^ = 0.0%, Figure 3F). Among the 12 cytokine indicators included, only RANTES showed a non-significant SMD, as detailed in the Tables S5 and S6. The above indicators are detailed in Figure 2 and Figure 3.

### 3.3. Subgroup Analysis

Given the high heterogeneity observed in the overall effect estimates for most indicators, we chose to use subgroup analysis to explore the sources of this heterogeneity. We specifically considered the following variables: patient groups (mild vs. severe cases, survivors vs. non-survivors), average age, sex ratio, study period, sample size, and study time. Subsequently, we conducted subgroup analyses for different cytokine indicators.

The subgroup analysis of IL-6 showed that the subgroup with average age ≥65 years (SMD = 1.12, 95% CI: 0.94–1.31, I^2^ = 33.5%, Figure S1B) exhibited lower heterogeneity relative to the pooled SMD and its 95% CI.

For IL-8, subgroup analyses based on patient groups (mild vs. severe cases) (SMD = 0.74, 95% CI: 0.41–1.08, I^2^ = 55.8%, Figure S2A), study period ≥20 months (SMD = 0.83, 95% CI: 0.56–1.09, I^2^ = 54.8%, Figure S2D), and study time ≥2025 (SMD = 0.97, 95% CI: 0.60–1.35, I^2^ = 56.6%, Figure S2E) all showed lower heterogeneity compared to the overall SMD and 95% CI.

For IL-10, subgroups including patient groups (mild vs. severe cases) (SMD = 1.03, 95% CI: 0.83–1.22, I^2^ = 0.0%, Figure S3A), average age ≥65 years (SMD = 1.17, 95% CI: 0.92–1.42, I^2^ = 39.8%, Figure S3B), male–female sex ratio <1 (SMD = 1.11, 95% CI: 0.96–1.27, I^2^ = 6.1%, Figure S3C), study period from 2020 to 2025 (SMD = 1.05, 95% CI: 0.81–1.29, I^2^ = 11.7%, Figure S3E), and sample size ≥90 (SMD = 1.15, 95% CI: 0.99–1.31, I^2^ = 20.0%, Figure S3F) all demonstrated lower heterogeneity compared to the overall SMD and 95% CI.

For IL-1β, subgroups with average age <65 years (SMD = 0.36, 95% CI: 0.11–0.60, I^2^ = 0.0%, Figure S4B), study period ≥20 months (SMD = 0.49, 95% CI: 0.24–0.75, I^2^ = 46.7%, Figure S4C), and sample size ≥90 (SMD = 0.49, 95% CI: 0.24–0.75, I^2^ = 46.7%, Figure S4E) showed lower heterogeneity compared to the overall SMD and 95% CI.

For TNF-α, the subgroup with a male–female sex ratio <1 (SMD = 0.74, 95% CI: 0.51–0.97, I^2^ = 47.9%, Figure S5C) exhibited lower heterogeneity compared to the overall SMD and 95% CI.

For IFN-γ, the subgroup with a male–female sex ratio ≥1 (SMD = 0.34, 95% CI: −0.11–0.79, I^2^ = 45.2%, Figure S6C) showed lower heterogeneity compared to the overall SMD and 95% CI.

For IP-10, the subgroup with a male–female ratio ≥1 (SMD = 0.76, 95% CI: 0.35–1.16, I^2^ = 0.0%, Figure S7A) and the subgroup with sample size ≥50 (SMD = 1.02, 95% CI: 0.59–1.44, I^2^ = 13.1%, Figure S7C) both demonstrated lower heterogeneity relative to the pooled SMD and its 95% CI.

### 3.4. Publication Bias and Sensitivity Analysis

**Figure 4:**
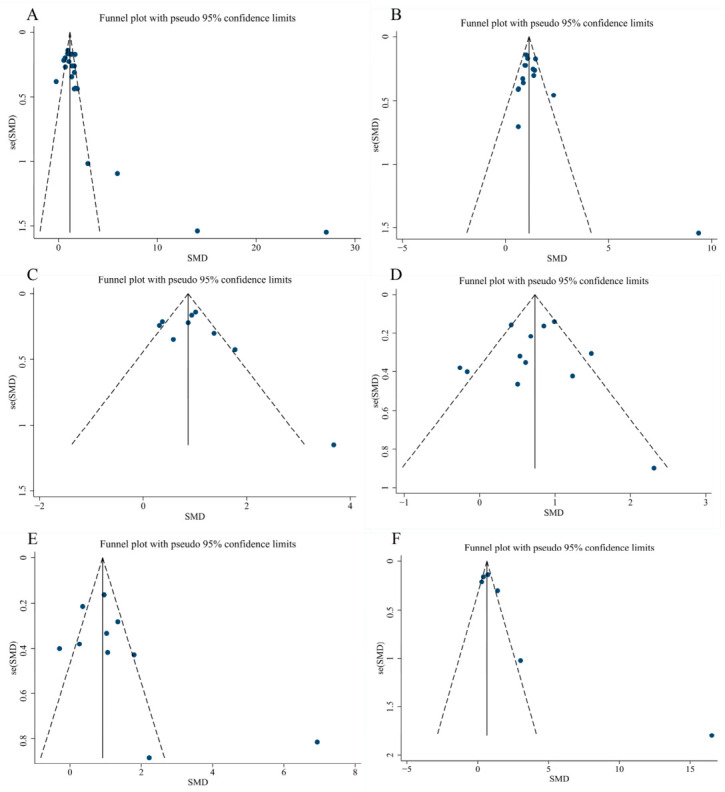
Funnel plot of cytokine indicators. (**A**) (IL-6), (**B**) (IL-10), (**C**) (IL-8), (**D**) (TNF-α), (**E**) (IFN-γ) and (**F**) (IL-1β).

**Figure 5:**
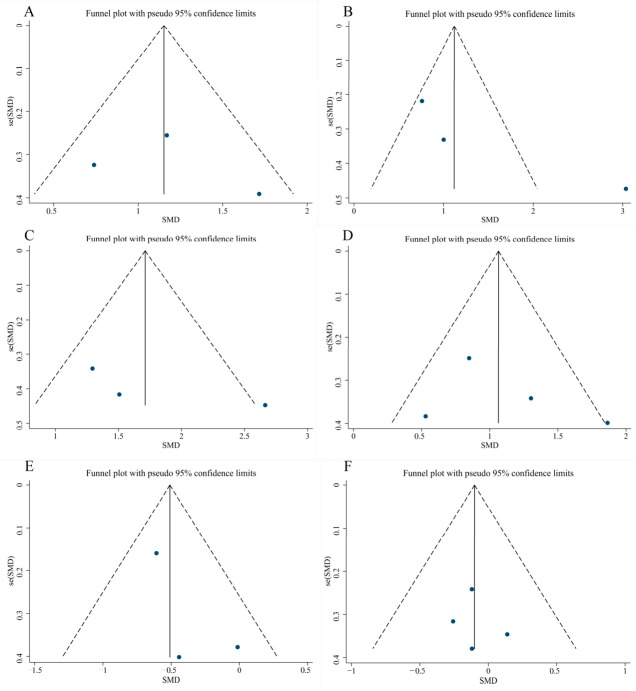
Funnel plot of cytokine indicators. (**A**) (MCP-1), (**B**) (IFN-α), (**C**) (G-CSF), (**D**) (IP-10), (**E**) (TGF-β), and (**F**) (RANTES).

**Figure 6:**
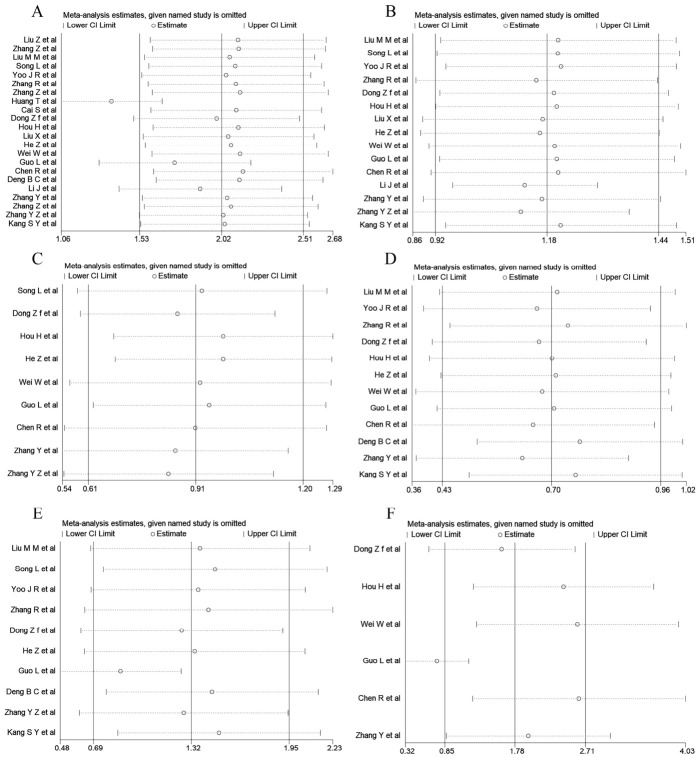
Sensitivity analysis of cytokine indicators. (**A**) (IL-6), (**B**) (IL-10), (**C**) (IL-8), (**D**) (TNF-α), (**E**) (IFN-γ) and (**F**) (IL-1β).

**Figure 7:**
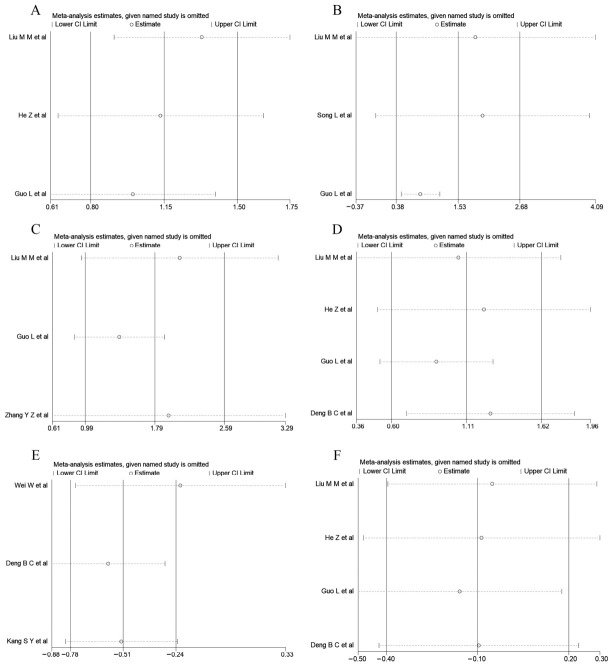
Sensitivity analysis of cytokine indicators. (**A**) (MCP-1), (**B**) (IFN-α), (**C**) (G-CSF), (**D**) (IP-10), (**E**) (TGF-β), and (**F**) (RANTES).

We used funnel plots and Egger’s and Begg’s tests to assess publication bias in the SMD and 95% CI estimates for 12 cytokine markers, including IL-6, IL-8, and IL-10. The results of Begg’s and Egger’s tests are shown in Tables S3 and S4, while the funnel plot results are presented in Figure 4 and Figure 5. The analysis revealed publication bias for IL-6, whereas no significant publication bias was observed for the remaining 11 cytokine markers. Subsequently, we performed sensitivity analyses to assess the stability and reliability of the SMD and its corresponding 95% CI across the 12 cytokine markers. The results confirmed that the findings were robust for all cytokines except IFN-α and TGF-β (Figure 6 and Figure 7).

## 4. Discussion

The acute phase constitutes a critical window in SFTS pathogenesis, characterized by the highest levels of viral replication and circulating viral load. During this phase, SFTSV deploys a dual-pronged immune evasion strategy: first, it actively suppresses type I interferon (IFN-I) production to evade innate antiviral immunity; second, it directly hijacks TBK1 to disrupt critical negative feedback loops that normally constrain inflammatory responses [39,40]. Consequently, the NF-κB pathway undergoes sustained, dysregulated activation, driving excessive production of pro-inflammatory cytokines, while concurrently impairing immune cell function and promoting aberrant macrophage and T-cell polarization [39,40]. This emergent pathological network drives a self-sustaining, escalating feedback loop that culminates in uncontrolled cytokine release and widespread multi-organ dysfunction [41]. Cytokine storm during the acute phase of SFTS is widely regarded as the principal driver of disease-associated mortality and morbidity in SFTSV infection [42]. Therefore, identifying cytokine markers capable of reliably stratifying SFTS severity is critically important. In this systematic review and meta-analysis, we comprehensively evaluated the associations between circulating levels of 12 key cytokines and clinical disease severity in SFTS patients.

Our meta-analysis identifies IL-6, IL-10, IP-10, and MCP-1 as cytokines with robust, statistically supported associations with SFTS severity. Numerous studies are consistent with our findings, showing that in critically ill patients, serum levels of IL-6, IL-10, IP-10, and MCP-1 are markedly increased and positively correlated with viral load [8,43]. Notably, in fatal cases, these cytokines not only reach higher peak concentrations but also demonstrate prolonged elevation, suggesting sustained immune dysregulation rather than transient inflammation [8,43]. In the study by Kwon J S et al., IL-6 is a pro-inflammatory cytokine and a key cytokine responsible for mortality in cytokine release syndrome [44]. Laboratory tests also showed significantly elevated levels of the inflammatory cytokines IL-6, IL-10, and TNF-α in SFTS patients [45,46]. Numerous studies have also reported significantly elevated levels of IL-10 in the serum of patients with fatal SFTS, severe COVID-19, as well as those infected with Ebola and H5N1 [47,48]. IP-10 concentrations correlate strongly with both viral load and clinical severity in SFTS [41]. Moreover, persistently elevated IP-10 is consistently associated with peripheral blood natural killer (NK) cell lymphopenia, underscoring its utility as a mechanistically grounded and clinically validated prognostic biomarker [49]. Increased MCP-1 levels in the cerebrospinal fluid may be potentially implicated in encephalopathy or encephalitis secondary to SFTS, suggesting that the virus might promote replication within the brain by disrupting the blood–brain barrier [32,50]. 

Our meta-analysis identifies TNF-α and IL-1β as robust, quantitatively associated biomarkers of disease severity in SFTS. TNF-α is predominantly produced by activated macrophages and antigen-experienced T lymphocytes and exhibits sustained overexpression in patients with severe or critical SFTS [51]. It induces the transcription of multiple pro-inflammatory genes via activation of the NF-κB and MAPK signaling pathways. Notably, after binding to TNFR1, TNF-α activates the caspase cascade, directly inducing apoptosis and leading to massive hepatocyte death, which in turn causes liver failure [52]. Furthermore, TNF-α acts cooperatively with IL-6 and IFN-γ to enhance leukocyte binding to vascular endothelium, thereby intensifying microvascular permeability, intravascular clot formation, and parenchymal damage [53]. A self-reinforcing interplay between TNF-α and IL-6 constitutes a central driver underlying the escalation of cytokine storm [53]. SFTS virus activates the NLRP3 inflammasome via multiple mechanisms, and it promotes the maturation and release of IL-1β [54]. Although a moderate elevation of IL-1β in the later stages may support antiviral immunity, pathologically elevated IL-1β during the acute phase drives uncontrolled inflammatory propagation and contributes directly to multi-organ injury [55,56].

IL-8, IFN-γ, IFN-α, and G-CSF may serve as valuable clinical biomarkers for assessing disease severity and thereby assist in developing personalized treatment strategies. It is well established that dysregulated secretion of cytokines and chemokines from both activated immune cells and virally infected host cells triggers pathological immune responses, ultimately leading to systemic multi-organ failure. In the study by Kwon J S et al., significantly elevated levels of IFN-α, IL-10, IP-10, IFN-γ, IL-6, IL-8, MCP-1, MIP-1α, G-CSF, and viral RNA were observed in plasma samples from fatal cases of SFTS, indicating that excessive cytokine release and uncontrolled viremia are key factors underlying these lethal outcomes [44]. In the study by Sun Y et al., plasma concentrations of IL-1RA, IL-10, IL-6, MCP-1, and G-CSF were significantly elevated in patients with severe SFTS compared with those with non-severe disease [8]. Similarly, multiple independent studies have consistently demonstrated that elevated circulating levels of IL-1β, IFN-γ, and IL-8 correlate with both disease progression and clinical severity in SFTS patients [6,57].

Our findings demonstrate that reduced circulating concentrations of TGF-β are significantly associated with increased disease severity in SFTS patients. Literature review indicates that TGF-β displays distinct temporal dynamics and prognostic stratification during the acute phase of SFTS: in the early disease stage, circulating TGF-β concentrations are significantly lower in patients who subsequently die compared with those who survive; conversely, among severely ill patients who ultimately recover, TGF-β levels undergo repeated, pronounced elevation and remain persistently elevated throughout the clinical course [58,59]. Diminished TGF-β levels during the initial phase of illness impair immunoregulation, permitting excessive inflammatory responses; conversely, elevated TGF-β in the advanced stage promotes anti-inflammatory signaling, facilitating inflammation resolution and restoration of tissue integrity [58,59]. The dynamic changes in TGF-β levels are strongly associated with disease prognosis, making it a crucial dynamic biomarker. Our study results indicate that the reduction in circulating RANTES levels is not significantly associated with increased disease severity in patients with SFTS. However, in some studies, RANTES showed a downregulation trend in SFTS patients [8], with significantly reduced expression and lower levels of RANTES, which may impair the recruitment of T cells and monocytes and delay viral clearance [60].

Our meta-analysis revealed substantial heterogeneity across most cytokine indicators, prompting rigorous subgroup analyses to identify potential sources. Key contributors identified included clinical stratification (mild vs. severe disease; survivors vs. non-survivors), demographic characteristics (mean age and sex ratio), and methodological factors (study period, sample size, and study time).

However, this study has several limitations. First, our analysis centered predominantly on cytokine indicators, with limited integration of clinical manifestations, routine laboratory parameters, host risk factors, pathogen characteristics, and other immunological mediators. Second, some articles present cytokine data in the form of figures without specific numerical values, making it impossible for us to conduct a systematic analysis of such studies. Finally, the sensitivity analyses for TGF-β and IFN-α yielded relatively unstable estimates due to the small number of eligible studies, underscoring the need for cautious interpretation. Despite these limitations, this study still provides foundational data supporting further clinical research, and understanding these cytokine markers will offer rational explanations for the intervention and treatment of SFTS.

## 5. Conclusions

In summary, this meta-analysis identified cytokines upregulated in critically ill patients, including IL-6, IL-10, IL-8, IFN-γ, TNF-α, MCP-1, IL-1β, IFN-α, G-CSF, and IP-10, indicating that elevated levels of these cytokines are associated with disease severity. Cytokines downregulated in critically ill patients included TGF-β and RANTES. These findings could serve as potential biomarkers for assessing patient condition and predicting prognosis, and provide a basis for developing targeted cytokine therapy strategies to alleviate immune-mediated tissue damage and improve survival rates.

## Figures and Tables

**Table 1:** Characteristics of the included studies of SFTS.

Author	Study Location	Study Period	Mean Age	Male%	Sample Size	NOS Score
Liu Z et al.	Yantai, China	2020.01–2021.12	67.00	47.28%	184	5
Zhang Z et al.	Wuhan, China	2016.08–2023.10	NR	48.78%	246	7
Liu M M et al.	NR	NR	61.20	34.00%	50	7
Song L et al.	Shandong, China	2021.04–2023.08	65.05	52.08%	96	9
Yoo J R et al.	Jeju Island	2013.04–2019.12	62.80	57.41%	54	7
Zhang R et al.	Yantai, China	2022.01–2024.12	66.00	48.84%	215	7
Zhang Z et al.	Wuhan, China	2016.08–2023.06	65.00	52.88%	208	6
Huang T et al.	Nanjing, China	2021.01–2022.12	NR	50.96%	157	6
Cai S et al.	Nanjing, China	2024.04–2024.07	NR	46.67%	60	6
Dong Z f et al.	Suzhou, China	2023.04–2024.08	66.80	30.00%	10	7
Hou H et al.	Wuhan, China	2021.04–2023.05	64.00	40.86%	93	8
Liu X et al.	Anhui, China	2019.09–2023.06	62.83	46.32%	95	7
He Z et al.	Xinyang, China	2018.05–2019.07	65.45	50.00%	100	9
Wei W et al.	Shanghai, China	2022.03–2024.06	63.87	42.26%	168	8
Guo L et al.	Nanjing, China	2023.05–2024.10	NR	45.65%	46	7
Chen R et al.	Wuhan, China	2022.03–2024.08	65.00	47.29%	277	6
Deng B C et al.	Dongbei, China	2011.04–2011.11	55.76	72.50%	40	8
Li J et al.	Nanjing, China	2011.05–2013.07	NR	54.17%	24	5
Zhang Y et al.	Anhui, China	2020.04–2020.12	NR	54.55%	77	8
Zhang Z et al.	Wuhan, China	2016.08–2023.07	NR	50.69%	290	6
Zhang Y Z et al.	Wuhan, China	2010.04–2010.10	54.40	NR	49	8
Kang S Y et al.	Jeju Island	2013.05–2022.04	63.70	56.92%	65	8

NR: not reported.

## Data Availability

All the data analyzed in this study are available in publicly available databases.

## Supplementary Materials

The following supporting information can be downloaded at: https://www.mdpi.com/article/10.3390/pathogens15070755/s1. Table S1: Search strategy in different databases; Table S2: PRISMA 2020 Checklist; Table S3: Begg’s test results of the final inclusion indicators; Table S4: Egger’s test results of the final inclusion indicators; Table S5: Mean ± SD of Cytokines extracted from the included studies. Table S6: Forest plot analysis results of the included cytokines. Table S7: Formulas for estimating the sample mean and standard deviation based on sample size and interquartile range. Figure S1: Subgroup analysis results of IL-6. A (Patient group), B (Average age), C (Male-female sex ratio), D (Study period), E (Time) and F (Sample size); Figure S2: Subgroup analysis results of IL-8. A (Patient group), B (Average age), C (Male-female sex ratio), D (Study period), E (Time) and F (Sample size); Figure S3: Subgroup analysis results of IL-10. A (Patient group), B (Average age), C (Male-female sex ratio), D (Study period), E(Time) and F (Sample size); Figure S4: Subgroup analysis results of IL-1β. A (Patient group), B (Average age), C (Study period), D(Time) and E (Sample size); Figure S5: Subgroup analysis results of TNF-α. A (Patient group), B (Average age), C (Male-female sex ratio), D (Study period), E(Time) and F (Sample size); Figure S6: Subgroup analysis results of IFN-γ. A (Patient group), B (Average age), C (Male-female sex ratio), D (Study period), E (Time) and F (Sample size); Figure S7: Subgroup analysis results of IP-10. A (Male-female sex ratio), B (Time) and C (Sample size).
