# Supplementary material for: Cytokine Indicators Associated with Disease Severity in Severe Fever with Thrombocytopenia Syndrome: A Systematic Review and Meta-Analysis
Source: Pathogens. 2026 Jul 17;15(7):755. doi: 10.3390/pathogens15070755 (PMC13415233; doi:10.3390/pathogens15070755)
Supplement: Supplementary file 1 [file pathogens-15-00755-s001.zip › pathogens-4391046-supplementary/pathogens-4391046-supplementary.pdf]

Table S1. Search strategy in different databases.

Search terms for PubMed (n =188), until 7 April 2026
#1 (("Severe Fever with Thrombocytopenia Syndrome"[Mesh] OR "Severe Fever with Thrombocytopenia Syndrome"[tiab] OR "SFTS"[tiab] OR "SFTSV"[tiab] OR "Huaiyangshan virus"[tiab] OR "Dabie bandavirus"[Mesh] OR "Dabie bandavirus"[tiab]))
#2 (("Cytokines"[Mesh] OR "Cytokine"[tiab] OR "Chemokines"[Mesh] OR "Chemokine"[tiab] OR "Interleukins"[Mesh] OR "Interleukin*"[tiab] OR "IL-"[tiab] OR "Tumor Necrosis Factor-alpha"[Mesh] OR "TNF-alpha"[tiab] OR "TNF-α"[tiab] OR "Interferon-gamma"[Mesh] OR "IFN-gamma"[tiab] OR "IFN-γ"[tiab] OR "Chemokine CXCL10"[tiab] OR "IP-10"[tiab] OR "Inflammation Mediators"[Mesh]))
(#1 And #2)
Search terms for Web of Science (n = 276), until 7 April 2026
#1 TS= (("Severe Fever with Thrombocytopenia Syndrome" OR SFTS OR SFTSV OR "Huaiyangshan virus" OR "Dabie bandavirus"))
#2 TS= ((Cytokine* OR Chemokine* OR Interleukin* OR "IL-*" OR "TNF-*" OR "Tumor Necrosis Factor*" OR "IFN-*" OR Interferon* OR "IP-10" OR CXCL10 OR "Inflammatory mediator*"))
(#1 And #2)
Search terms for Embase (n = 604), until 7 April 2026
#1 (('severe fever with thrombocytopenia syndrome'/exp OR 'severe fever with thrombocytopenia syndrome':ab,ti OR 'sfts':ab,ti OR 'sftsv':ab,ti OR 'huaiyangshan virus':ab,ti OR 'dabie bandavirus':ab,ti))
#2 (('cytokine'/exp OR 'cytokine*':ab,ti OR 'chemokine'/exp OR 'chemokine*':ab,ti OR 'interleukin'/exp OR 'interleukin*':ab,ti OR 'il*':ab,ti OR 'tumor necrosis factor'/exp OR 'tnf*':ab,ti OR 'interferon'/exp OR 'interferon*':ab,ti OR 'ifn*':ab,ti OR 'cxcl10':ab,ti OR 'ip-10':ab,ti))
(#1 And #2)

Table S3. Begg's test results of the final inclusion indicators.

Cytokine	Number of Studies	Begg's Test	
		z	*P*
IL-6	22	3.10	0.002
IL-8	9	1.15	0.251
IL-10	15	0.49	0.621
TNF-α	12	0.34	0.732
IFN-γ	10	1.25	0.210
IL-1β	6	1.13	0.260
IP-10	4	0.34	0.734
MCP-1	3	0.00	1.000
G-CSF	3	1.04	0.296
IFN-α	3	1.04	0.296
TGF-β	3	0.00	1.000
RANTES	4	0.34	0.734

Table S4. Egger's test results of the final inclusion indicators.

Cytokine	Number of Studies	Egger's Test	
		t	*P*
IL-6	22	3.64	0.002
IL-8	9	1.03	0.337
IL-10	15	1.41	0.181
TNF-α	12	-0.23	0.821
IFN-γ	10	1.54	0.162
IL-1β	6	2.83	0.047
IP-10	4	0.69	0.563
MCP-1	3	0.43	0.739
G-CSF	3	1.45	0.385
IFN-α	3	2.14	0.278
TGF-β	3	1.40	0.395
RANTES	4	0.37	0.745

Table S5. Mean ± SD of Cytokines extracted from the included studies.

Cytokine	Study	Mild patients	Severe patients
IL-6	Liu Z et al	34.1285±39.6018	163.5393±186.0835
	Zhang Z et al	32.4897±31.0961	55.7516±54.3623
	Liu M M et al	24.3517±28.9292	148.6273±171.6672
	Song L et al	7.3855±8.6105	37.7341±46.4017
	Yoo J R et al	15.4677±26.5053	6269.555±11490.672
	Zhang R et al	17.9776±22.0695	107.4102±126.7673
	Zhang Z et al	45.0213±55.0931	238.8602±396.3271
	Huang T et al	21.6 ± 23.9	542.4 ± 1.822
	Cai S et al	17.8 ± 16.5	44.8 ± 55.3
	Dong Z f et al	1.78 ± 0.70	3.63 ± 0.27
	Hou H et al	118.70 ± 212.24	464.05 ± 987.15
	Liu X et al	15.7981±17.7657	120.8985±125.8655
	He Z et al	60.7130±54.4897	145.0409±82.4266
	Wei W et al	31.4961±33.7281	166.0416±216.8378
	Guo L et al	173.28 ± 16.36	2179.58 ± 297.96
	Chen R et al	34.8411±39.6455	113.7997±144.5718
	Deng B C et al	143.1856±276.0458	85.1645±114.6474
	Li J et al	2.85±6.06	1268.88±703.47
	Zhang Y et al	10.6942±8.5371	76.8082±90.3578
	Zhang Z et al	24.3196±16.6144	89.5355±86.6481
	Zhang Y Z et al	23.6080±32.2308	211.3039±242.3416
	Kang S Y et al	120.4111±246.9317	6264.166±11438.010
IL-8	Song L et al	4.6750±6.4202	19.8404±27.7131

	Dong Z f et al	1.94 ± 0.52	3.66 ± 0.25
	Hou H et al	214.07 ± 713.33	655.90 ± 1666.16
	He Z et al	66.3656±0.6469	66.5750±0.7447
	Wei W et al	23.9252±15.9649	70.8608±74.6834
	Guo L et al	1396.54 ± 146.78	1487.43 ± 184.68
	Chen R et al	23.4966±17.5871	97.2865±134.5859
	Zhang Y et al	22.7210±13.2212	115.7686±149.6645
	Zhang Y Z et al	15.3088±23.3711	107.2794±122.1255
IL-10	Liu M M et al	1240.546±1792.621	5084.445±8334.523
	Song L et al	4.7803±6.5181	21.8539±29.0603
	Yoo J R et al	28.1425±55.0671	66.8041±104.3457
	Zhang R et al	15.0548±19.2931	70.7484±68.4029
	Dong Z f et al	1.92 ± 0.55	2.22 ± 0.16
	Hou H et al	67.68 ± 100.77	232.73 ± 240.30
	Liu X et al	27.4171±33.8000	130.0072±140.0179
	He Z et al	20.3662±20.5578	92.8855±103.4207
	Wei W et al	44.5725±53.9141	131.5300±107.3795
	Guo L et al	65.76 ± 14.32	87.87 ± 46.85
	Chen R et al	42.8660±54.4752	133.0120±144.1992
	Li J et al	2.97±3.53	48.089±11.42
	Zhang Y et al	36.7051±40.1926	150.5963±167.4943
	Zhang Y Z et al	9.0079±18.7885	80.6363±65.5308
	Kang S Y et al	27.9210±54.5873	66.5157±104.3982
IFN-γ	Liu M M et al	12.8345±16.1821	50.0532±64.2702
	Song L et al	39.9075±65.6624	78.6169±157.8937

	Yoo J R et al	280.3368±517.6157	1310.565±2482.057
	Zhang R et al	120.5365±155.0539	336.4674±359.1430
	Dong Z f et al	1.65 ± 0.75	3.12 ± 0.25
	He Z et al	30.7618±28.3078	123.4503±122.1555
	Guo L et al	32.25 ± 5.39	70.34 ± 5.83
	Deng B C et al	553.624±1176.847	892.3155±1623.2879
	Zhang Y Z et al	26.5317±49.2626	277.3411±342.1653
	Kang S Y et al	275.8256±515.2434	131.6368±227.5987
TNF-α	Liu M M et al	1.1646±0.6870	1.9724±2.6716
	Yoo J R et al	2.8631±5.7885	20.5410±39.0725
	Zhang R et al	1.5386±0.9628	1.9930±1.3807
	Dong Z f et al	1.82 ± 0.45	2.77 ± 0.26
	Hou H et al	40.64 ± 41.27	162.04 ± 280.02
	He Z et al	84.6639±125.2698	172.7717±231.4870
	Wei W et al	22.5280±14.3198	49.2883±45.3335
	Guo L et al	91.87 ± 7.89	98.67 ± 18.30
	Chen R et al	23.0886±13.4884	49.4335±45.3091
	Deng B C et al	96.4878±176.7770	54.8689±58.9000
	Zhang Y et al	19.1051±6.9506	64.6036±67.2395
	Kang S Y et al	2.8298±5.7620	1.9178±3.4875
IL-1β	Dong Z f et al	-0.31 ± 0.46	0.90 ± 0.08
	Hou H et al	18.17 ± 44.02	31.88 ± 57.80
	Wei W et al	6.9489±4.1501	9.4257±8.1881
	Guo L et al	39.18 ± 4.67	244.86 ± 24.64
	Chen R et al	5.9820±2.0866	8.9341±7.1541

	Zhang Y et al	8.9108±6.4217	23.2288±18.9630
IP-10	Liu M M et al	2397.56±1873.76	5734.216±3840.949
	He Z et al	972.6906±1020.1403	1835.5806±998.8755
	Guo L et al	8292.39 ± 972.84	10080.67 ± 909.67
	Deng B C et al	210.8227±231.1403	362.3541±432.2155
MCP-1	Liu M M et al	191.9184±194.2610	528.0488±814.2933
	He Z et al	163.0345±115.5236	657.4525±886.1350
	Guo L et al	141.02 ± 27.24	186.04 ± 22.51
G-CSF	Liu M M et al	7.6432±4.0455	27.3903±28.5476
	Guo L et al	7.83 ± 2.54	16.71 ± 5.19
	Zhang Y Z et al	27.2982±41.0140	133.2670±153.5925
IFN-α	Liu M M et al	11.7432±10.3046	61.8653±94.6498
	Song L et al	9.5406±18.4101	69.9686±130.1355
	Guo L et al	14.11 ± 6.86	35.07 ± 7.06
TGF-β	Wei W et al	29.5701±54.2334	4.4591±3.4913
	Deng B C et al	42.5958±74.6631	41.6003±79.2767
	Kang S Y et al	748.4293±1252.2478	220.6491±298.7896
RANTES	Liu M M et al	5244.407±3564.480	4385.403±2648.972
	He Z et al	1243.5495±679.2561	1165.3393±545.0627
	Guo L et al	4448.42 ± 98.67	4461.56 ± 78.62
	Deng B C et al	1726.863±2246.198	1469.653±1819.596

Table S6. Forest plot analysis results of the included cytokines.

Cytokine	Test of SMD	
	z	*P*
IL-6	8.08	0.000
IL-8	5.94	0.000
IL-10	8.79	0.000
TNF-α	5.20	0.000
IFN-γ	4.11	0.000
IL-1β	3.75	0.000
IP-10	4.25	0.000
MCP-1	6.46	0.000
G-CSF	4.37	0.000
IFN-α	2.60	0.009
TGF-β	3.68	0.000
RANTES	0.66	0.510

Table S7. Formulas for estimating the sample mean and standard deviation based on sample size and interquartile range.

Variable	Formulas
Mean Estimation	(First quartile + Median + Third quartile)/3
Standard Deviation Estimation	(Third quartile - First quartile)/ (2* NORM. INV ((0.75*Sample Size-0.125)/ (Sample Size+0.25),0,1))

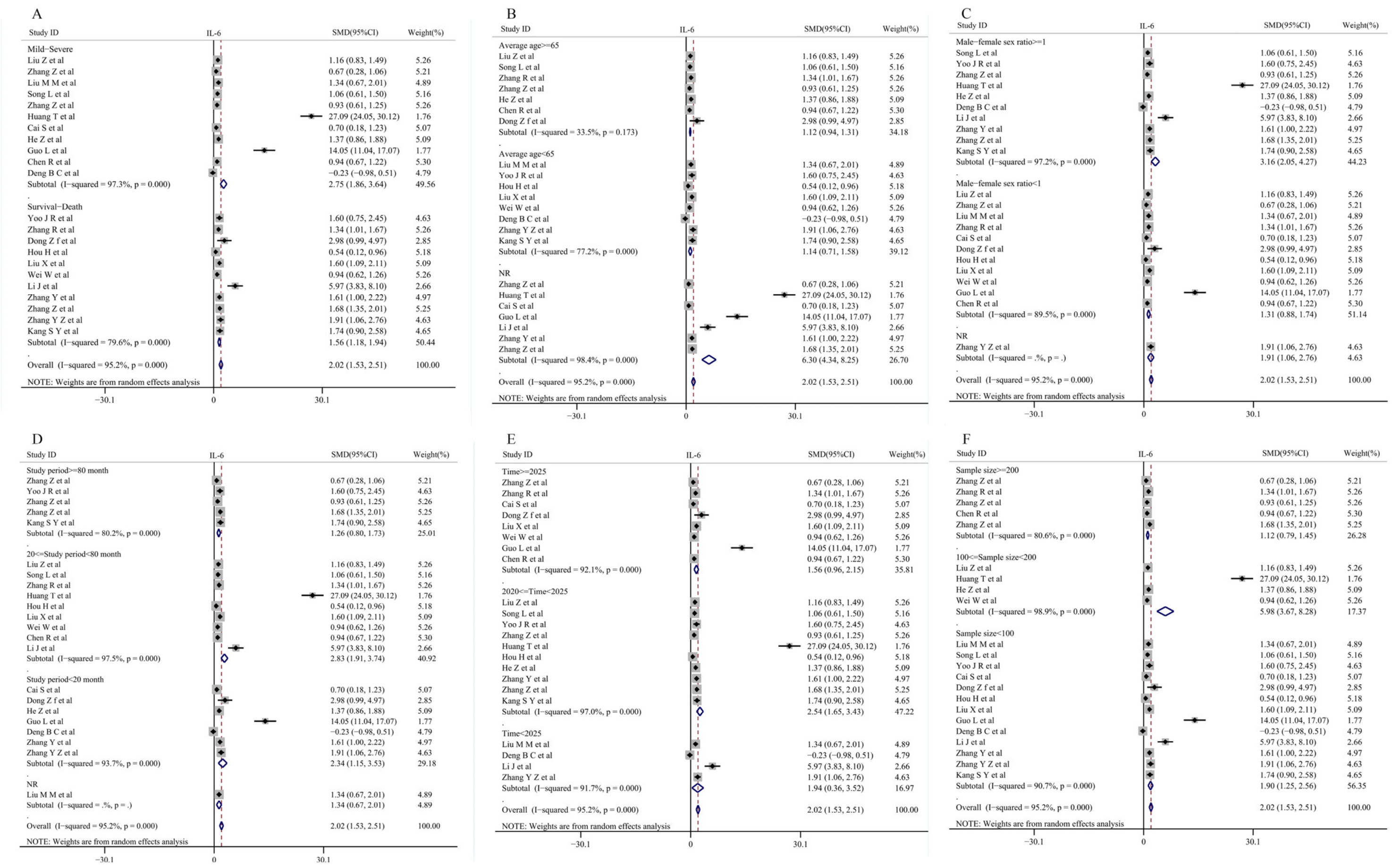

Figure S1. Subgroup analysis results of IL-6. A (Patient group), B (Average age), C (Male-female sex ratio), D (Study period), E (Time) and F (Sample size).

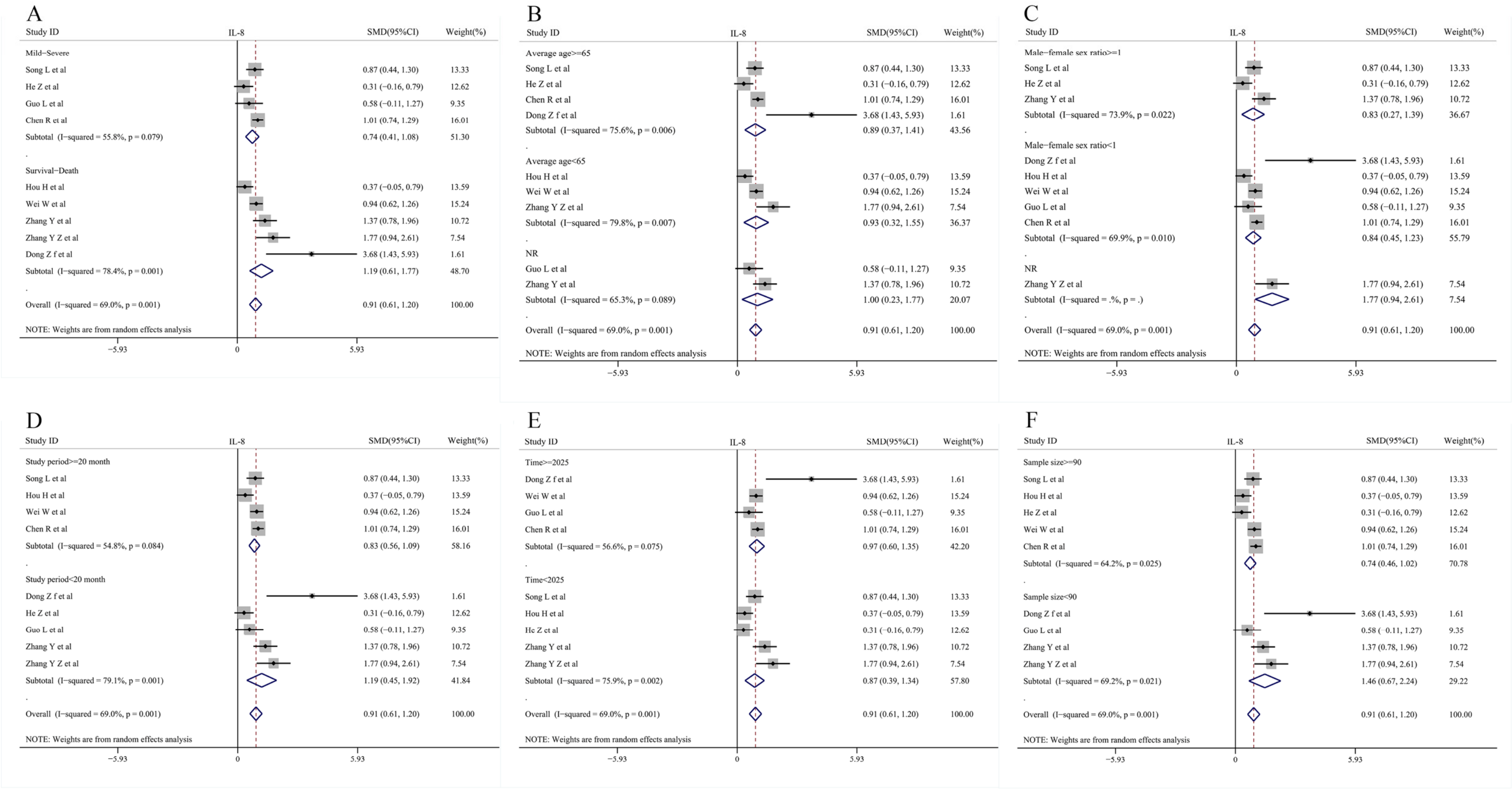

Figure S2. Subgroup analysis results of IL-8. A (Patient group), B (Average age), C (Male-female sex ratio), D (Study period), E (Time) and F (Sample size).

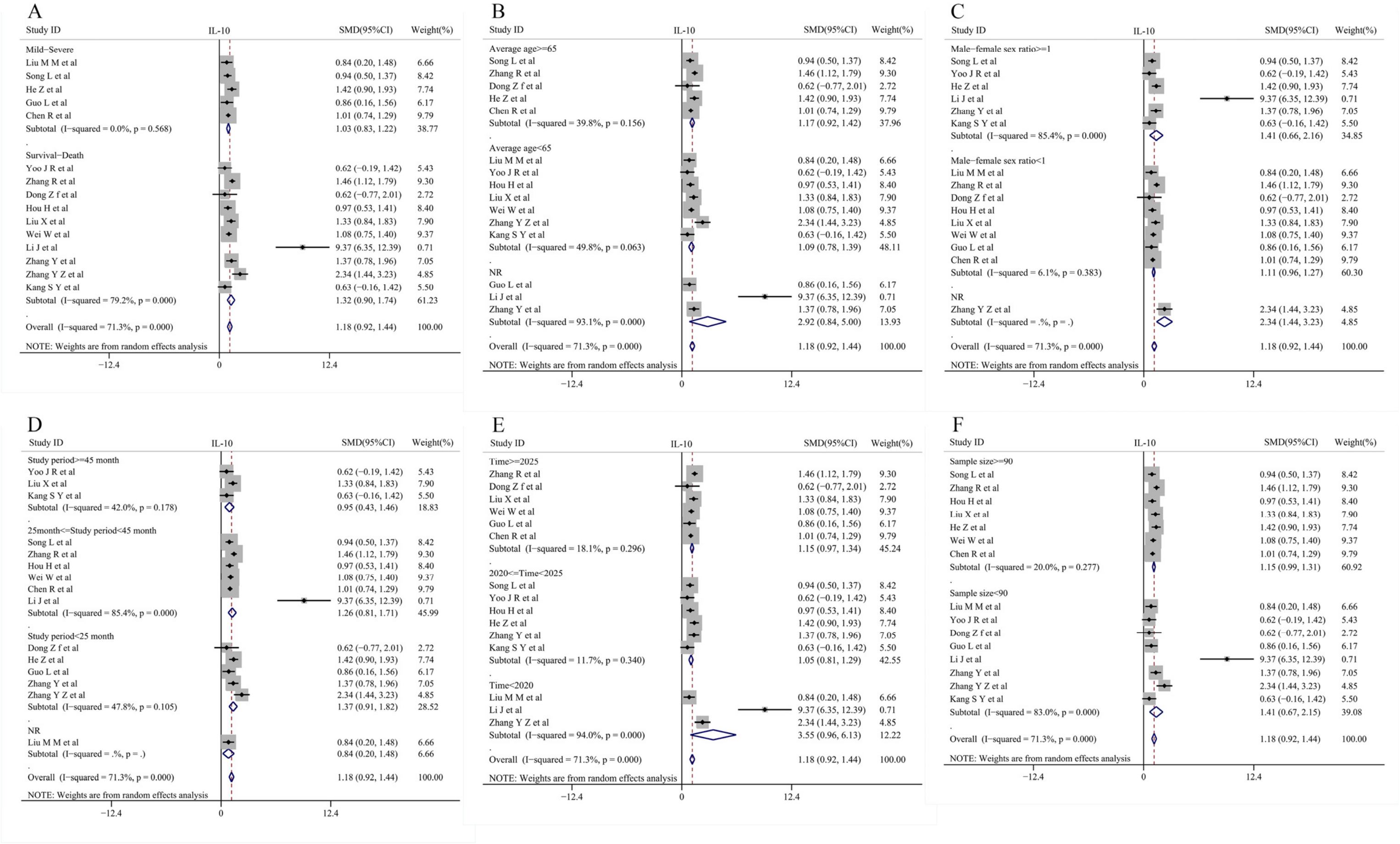

Figure S3. Subgroup analysis results of IL-10. A (Patient group), B (Average age), C (Male-female sex ratio), D (Study period), E(Time) and F (Sample size).

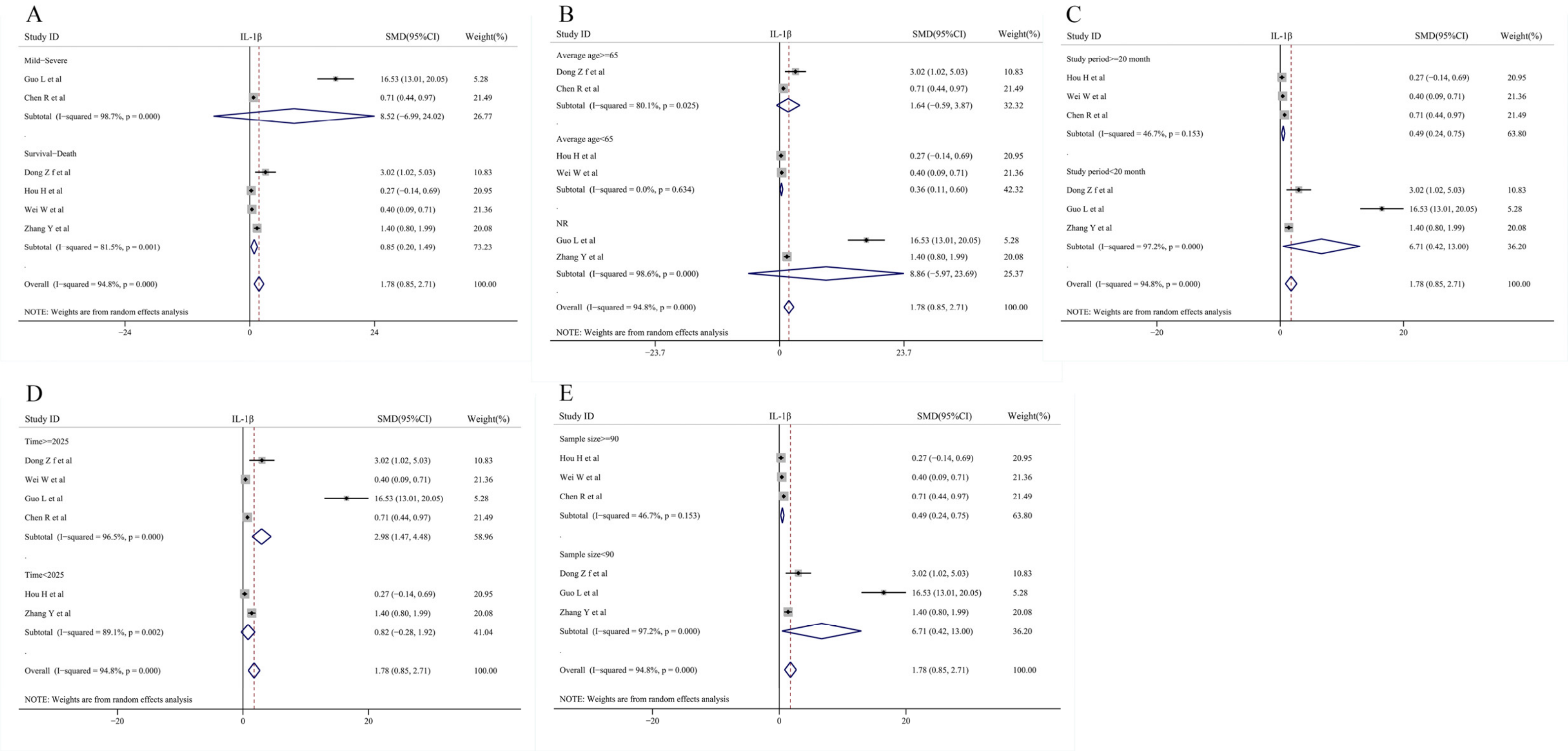

Figure S4. Subgroup analysis results of IL-1β. A (Patient group), B (Average age), C (Study period), D(Time) and E (Sample size).

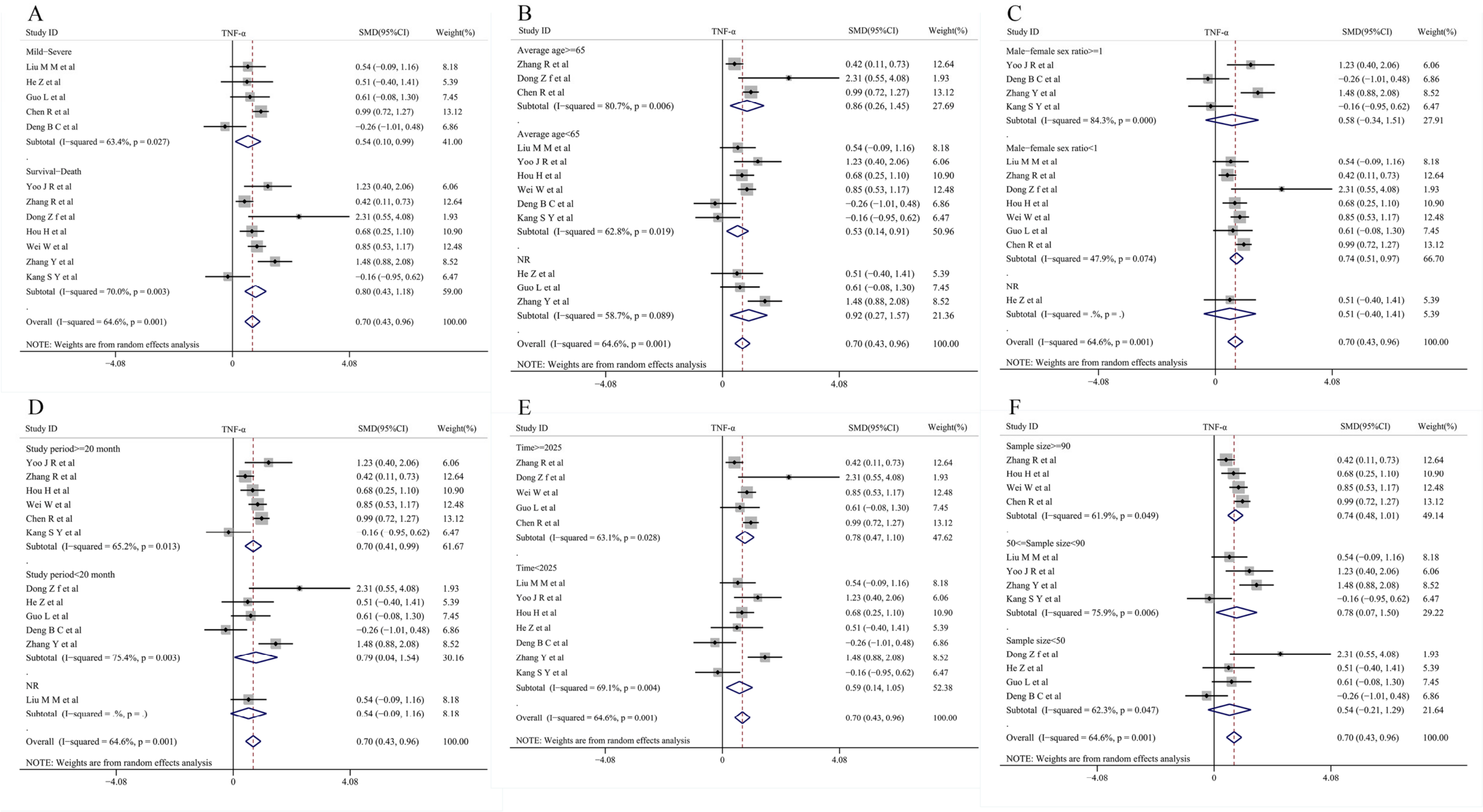

Figure S5. Subgroup analysis results of TNF-α. A (Patient group), B (Average age), C (Male-female sex ratio), D (Study period), E(Time) and F (Sample size).

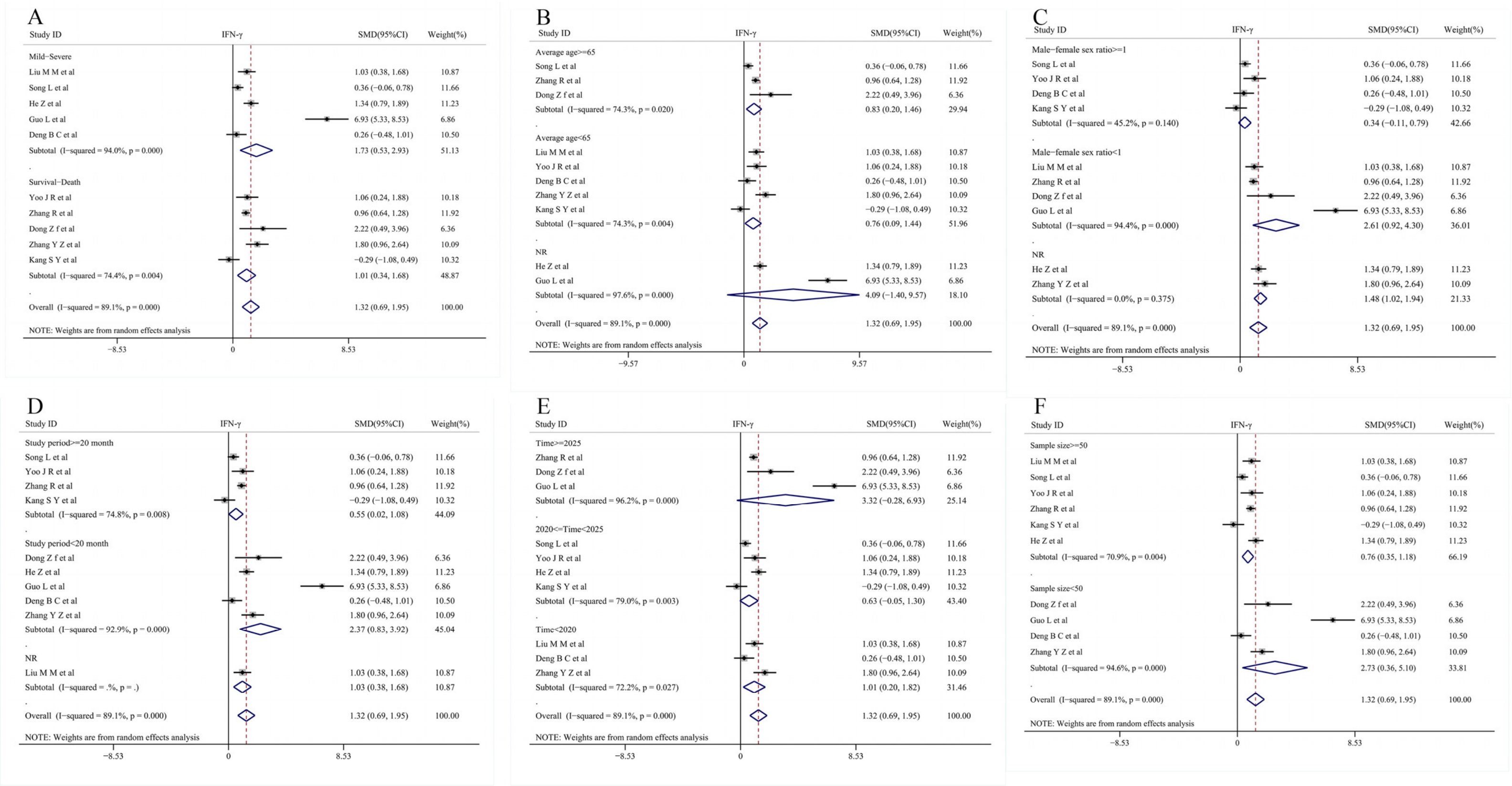

Figure S6. Subgroup analysis results of IFN-γ. A (Patient group), B (Average age), C (Male-female sex ratio), D (Study period), E (Time) and F (Sample size).

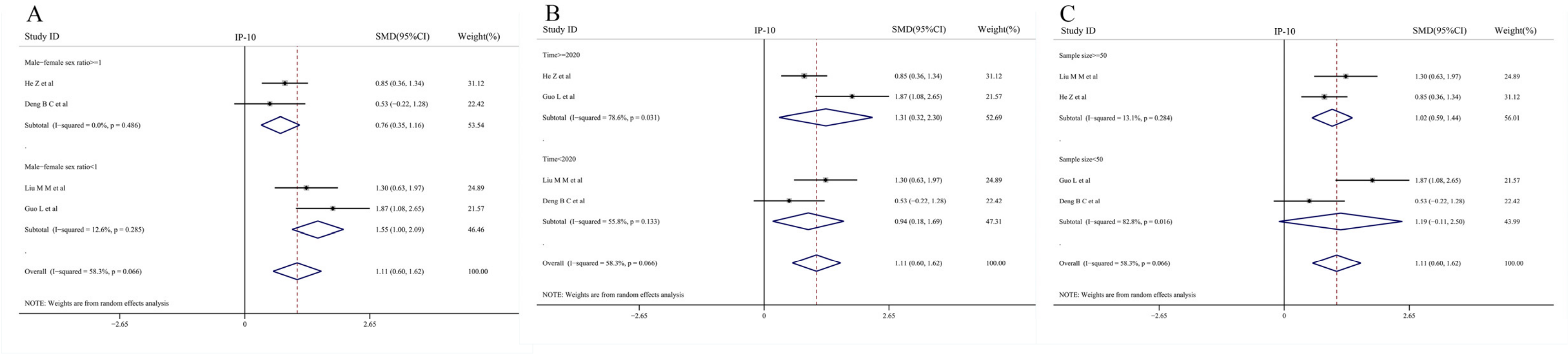

Figure S7. Subgroup analysis results of IP-10. A (Male-female sex ratio), B (Time) and C (Sample size).
